# Barriers and facilitators to reducing frequent laboratory testing for patients who are stable on warfarin: a mixed methods study of de-implementation in five anticoagulation clinics

**DOI:** 10.1186/s13012-017-0620-x

**Published:** 2017-07-14

**Authors:** Geoffrey D. Barnes, Sevan Misirliyan, Scott Kaatz, Elizabeth A. Jackson, Brian Haymart, Eva Kline-Rogers, Jay Kozlowski, Gregory Krol, James B. Froehlich, Anne Sales

**Affiliations:** 1 Frankel Cardiovascular Center, Department of Internal Medicine, University of Michigan Medical School, 2800 Plymouth Rd, B14 G101, Ann Arbor, MI 48109-2800 USA; 2 Institute for Healthcare Policy and Innovation, University of Michigan Medical School, Ann Arbor, MI USA; 3 Division of Hospital Medicine, Department of Internal Medicine, Henry Ford Hospital, Detroit, MI USA; 4 Cardiovascular Associates, Huron-Valley Sinai Hospital, Commerce Township, MI USA; 5 Department of Internal Medicine, Henry Ford Hospital, Detroit, MI USA; 6 Department of Learning Health Sciences, University of Michigan Medical School, Ann Arbor, MI USA; 7 VA Center for Clinical Management Research, VA Ann Arbor Healthcare System, Ann Arbor, USA

**Keywords:** Warfarin, Anticoagulation, Implementation, Quality improvement

## Abstract

**Background:**

Patients on chronic warfarin therapy require regular laboratory monitoring to safely manage warfarin. Recent studies have challenged the need for routine monthly blood draws in the most stable warfarin-treated patients, suggesting the safety of less frequent laboratory testing (up to every 12 weeks). De-implementation efforts aim to reduce the use of low-value clinical practices. To explore barriers and facilitators of a de-implementation effort to reduce the use of frequent laboratory tests for patients with stable warfarin management in nurse/pharmacist-run anticoagulation clinics, we performed a mixed-methods study conducted within a state-wide collaborative quality improvement collaborative.

**Methods:**

Using a mixed-methods approach, we conducted post-implementation semi-structured interviews with a total of eight anticoagulation nurse or pharmacist staff members at five participating clinic sites to assess barriers and facilitators to de-implementing frequent international normalized ratio (INR) laboratory testing among patients with stable warfarin control. Interview guides were based on the Tailored Implementation for Chronic Disease (TICD) framework. Informed by interview themes, a survey was developed and administered to all anticoagulation clinical staff (*n* = 62) about their self-reported utilization of less frequent INR testing and specific barriers to de-implementing the standard (more frequent) INR testing practice.

**Results:**

From the interviews, four themes emerged congruent with TICD domains: (1) staff overestimating their actual use of less frequent INR testing (individual health professional factors), (2) barriers to appropriate patient engagement (incentives and resources), (3) broad support for an electronic medical record flag to identify potentially eligible patients (incentives and resources), and (4) the importance of personalized nurse/pharmacist feedback (individual health professional factors). In the survey (65% response rate), staff report offering less frequent INR testing to 56% (46–66%) of eligible patients. Most survey responders (*n* = 24; 60%) agreed that an eligibility flag in the electronic medical record would be very helpful. Twenty-four (60%) respondents agreed that periodic, personalized feedback on use of less frequent INR testing would also be helpful.

**Conclusions:**

Leveraging information system notifications, reducing additional work load burden for participating patients and providers, and providing personalized feedback are strategies that may improve adoption and utilization new policies in anticoagulation clinics that focus on de-implementation.

**Electronic supplementary material:**

The online version of this article (doi:10.1186/s13012-017-0620-x) contains supplementary material, which is available to authorized users.

## Background

Patients on chronic warfarin therapy require regular international normalized ratio (INR) laboratory monitoring to measure the degree of anticoagulant effect and to adjust the dose of warfarin. After an initial period of frequent dose titration, many patients achieve a relative “steady state” where their warfarin dose does not fluctuate significantly [1]. Traditionally, most of these patients still received a INR blood draw at least every 4 weeks [2]. However, recent observational and prospective, randomized studies have challenged the need for such frequent blood draws in the most stable warfarin-treated patients, demonstrating safety with INR blood draws every 12 weeks [3, 4]. While supported by guidelines, adoption of a less frequent laboratory testing practice has not been widely adopted [2, 5].

Beginning in 2013, six anticoagulation clinics participating in an anticoagulation clinic quality improvement collaborative identified INR testing frequency as a quality improvement focus [6]. After extensive discussion at collaborative-wide quarterly meetings, individual anticoagulation clinics developed eligibility criteria for less frequent INR testing (Table 1). These criteria determined the number of INR tests that must be within the target range before a scheduled INR test could be extended beyond the traditional 4-week window. Based on these site-specific criteria, reporting tools were developed to provide real-time feedback to anticoagulation clinic staff about the utilization of less frequent INR testing (defined as a plan to re-check an INR ≥ 5 weeks from the most recent INR laboratory test). Overall rates of less frequent INR testing were reported and discussed at each subsequent quarterly meeting. However, utilization of this practice plateaued around 50–60% of eligible patients by the end of 2015.

**Table 1:** Extended INR testing policies by anticoagulation clinic

	Definition of “stable warfarin patient”	Maximal allowed INR testing interval
Clinic 1	12 weeks without maintenance dose change and no out of range INRs	10 weeks
Clinic 2	6 months without a weekly dose change and no INRs >10% above/below therapeutic range	12 weeks
Clinic 3	6 months without a weekly dose change and no INRs >10% above/below therapeutic range (both have to be met)	6 weeks
Clinic 4	10 weeks of no INRs > 10% above/below therapeutic range	6 weeks
Clinic 5	4 months without any INRs >10% out of range and no weekly dose change	6 weeks

The Tailored Implementation for Chronic Disease (TICD) framework was developed as a comprehensive, integrated checklist of determinants of implementation success, reconsolidating across 12 different reviews of implementation determinants [7]. By integrating elements from other commonly used frameworks, e.g., the Consolidated Framework for Implementation Science and the Theoretical Domains Framework, it aims to be an easily used checklist to identify determinants of practice for implementation strategy development and program evaluation [8, 9]. The TICD developers intended it to be used as a screening tool that can help identify implementation determinant that warrant further in-depth investigation and to facilitate tailoring of more effective change interventions and evaluation.

De-implementation, also known as de-adoption, is commonly defined as the reduction in use of low-value clinical practices [10]. As is highlighted by the recent Choosing Wisely campaign, many experts recommend against the routine use of common, but low-yield, clinical practices. These practices can cause unnecessary anxiety, harm, and cost for patients and the healthcare system [11]. Yet, specific barriers and facilitators to de-implementation of low-yield practices have not been thoroughly examined. And the use of implementation frameworks, such as the TICD framework, to evaluate de-implementation effort remains inadequately studied [10, 12].

The Michigan Anticoagulation Quality Improvement Initiative (MAQI^2^) is a collaborative of six anticoagulation clinics in Michigan aimed at improving the delivery of anticoagulation care with the support of Blue Cross Blue Shield of Michigan [13]. The collaborative is financially supported by the state-wide health insurer Blue Cross-Blue Shield of Michigan. The collaborative identifies important quality improvement efforts to work on jointly, shares best practices at quarterly meetings, and reviews chart abstracted data to monitor progress on the various quality improvement efforts. Patients newly starting on warfarin who are referred to one of the participating anticoagulation clinics are eligible to have their demographic and medical information abstracted into the MAQI^2^ registry. All data elements are manually abstracted from the medical chart by trained chart abstractors using pre-defined data elements. All INR lab tests, warfarin weekly doses, and communication between the anticoagulation clinic and the patient/family are abstracted into the registry.

We set out to explore barriers and facilitators for a de-implementation effort for patients with stable warfarin control who are managed at one of the anticoagulation clinics participating in MAQI^2^. The de-implementation effort (a new clinic policy) was targeted at anticoagulation clinic providers (nurses and pharmacists) with a goal of reducing frequent INR laboratory testing for patients with stable warfarin control. These nurses and pharmacists were asked to review each patients’ record for eligibility (i.e., stable warfarin management over sequential INR laboratory tests) to have less frequent INR laboratory tests. Each anticoagulation clinic developed their own specific policy outlining the specific eligibility criteria and maximal duration between scheduled INR laboratory tests (Table 1). This practice was to be carried out anytime an anticoagulation clinic staff member interacted with an eligible patient, which usually occurs when the staff member reviews the most recent INR laboratory test results and provides warfarin dosing recommendations to the patient.

Based on clinical experience, we hypothesized that technological barriers (e.g. difficulty identifying prior INR values in the electronic medical record) would exist to identifying appropriate patients for reduced INR testing frequency and that most anticoagulation clinic staff would underestimate their personal utilization of a less frequent INR testing.

## Methods

### Study design

We employed a mixed-methods approach for this study. As is recommended by the TICD authors, we reviewed the framework’s exhaustive checklist of twelve domains and 57 associated determinants of practice [7]. We selected the domains and determinants of practice that we felt were most relevant to the de-implementation of frequent INR laboratory testing among stable warfarin patients. From these domains and determinants, we developed an interview guide aimed at assessing specific barriers and facilitators to a de-implementation process (Additional file 1).

Based on the most salient themes that emerged from the interviews, a brief survey was developed (Additional file 1).

### Research setting and sampling strategy

Anticoagulation clinic staff (nurses and pharmacists) from all MAQI^2^ sites were invited to participate in the study. For this project, one of the MAQI^2^ sites elected to not participate in anticoagulation staff interviews or the survey, resulting in five participating sites. One or two nurses or pharmacist staff from the five participating MAQI^2^ sites participated in semi-structured interviews. The anticoagulation clinic leadership selected “representative” staff members (not necessarily the highest or lowest performing staff members). All nurses and pharmacists at the participating anticoagulation clinics (including those who participated in the interviews) were then invited to participate in the online survey.

### Data collection methods

After 2 years of consortium-wide discussion and review of INR testing frequency data, a mixed-methods study was undertaken to better understand the barriers and facilitators to less frequent INR testing for eligible patients on warfarin therapy. These interviews were conducted by two team members (GDB and SM) under the guidance of a third team member (AS) between May and July 2016. Interviews were conducted either in person or by phone.

All nursing and pharmacist staff at the five participating anticoagulation clinics were invited to anonymously complete a brief online survey. This survey assessed staff self-reported use of less frequent INR testing within the last 6 months (answer choice ranges of 0–20%, 21–40%, 41–60%, 61–80%, and 81–100% of the time), self-reported use of less frequent INR testing for the last 10 eligible patients, attitudes about periodic feedback, self-reported frequency checking eligibility (answer choices never, almost never, sometimes, almost every time, and every time), and perceived helpfulness of an electronic medical record flag for patient eligibility (answer choices not helpful, somewhat helpful, and very helpful). Survey invitations were sent on 29 August 2016 with one reminder e-mail sent on 7 September 2016.

### Data analysis methods

Interviews were audio recorded and study team members conducted framework analysis, coding into the TICD framework domains [14, 15]. Salient themes were identified by both team members who conducted the interviews (GDB and SM) and coded the transcripts. Frequency statistics were calculated for survey responses.

### Comparing survey self-response to actual performance data

To compare self-reported rates to actual rates of less frequent INR testing, we used data from the MAQI^2^ registry between January and June of 2016. Clinic-level rates of less frequent INR testing were calculated based on the individual anticoagulation clinic protocols (Table 1) and excluded any patients on home INR testing, with a ventricular assist device implanted, or who opted out of the less frequent INR testing practice. We used the midpoint of the survey response range (e.g., 50% for the survey response option of 40–60%) for point estimates. To generate confidence intervals (CI) around our summary point estimates, we used the lower and higher boundary from the survey response options (e.g., 40% and 60%) in repeat calculations.

### Regulatory review

Institutional review for patient data collection and analysis has been approved at all participating sites, including the University of Michigan (coordinating center). This project was reviewed and deemed “not regulated” by the University of Michigan Institutional Review Board since it did not meet regulatory definitions of “research” and instead was categorized as a quality improvement project.

## Results

Eight semi-structured interviews were conducted, seven with anticoagulation nurses, and one with a pharmacist. Four main themes emerged from these interviews. First, all interviewees reported being highly compliant with offering less frequent INR testing to eligible patients. They were surprised to hear that the actual overall utilization rates of less frequent INR testing for eligible patients were closer to 50–60% and not 80–100%, which they had predicted based on their own experience and belief about practice patterns. This linked with the TICD domain of individual health professional factors.

The second theme identified was variable barriers to appropriate patient engagement (TICD domain incentives and resources—which could be considered ‘dis-incentives and resources’ in this context). These barriers most often related to the individual health care center’s electronic medical record and/or policies. The first example centers on available data and ease of access to that data within the electronic medical record. Some centers offer simple flowsheets of INR results (at least 5–6) that are easy to review and identify trends. Other centers required multiple clicks within different computer screens to access these data. The second example of this barrier involves the prerequisites required before an anticoagulation clinic staff member can begin using a less frequent INR testing schedule for an individual patient. Some centers, but not all, use specialized processes or required extensive and additional patient education before allowing less frequent INR laboratory testing schedules. These two examples of barriers to appropriate patient engagement (inability to easily review INR labs and need for extra education or a separate process) were commonly cited, particularly on busier days in the anticoagulation clinic. Other interviewees identified a need for increased patient education, especially for patients with longer warfarin-treatment experience as such patients were often previously told that 4 weeks was the maximum interval between INR blood draws for safety reasons.

Third, the interviewees expressed broad support for an electronic medical record flag for potential extended INR testing eligibility (TICD domain incentives and resources). Interviewees acknowledged that while forgetting to look for eligibility was not the most common barrier, it likely contributed to overly frequent INR testing among patients with stable warfarin control.

Finally, all interviewees agreed the personalized provider-level feedback on use of less frequent INR testing for eligible patients would be very helpful (TICD domain individual health professional factor). It was noted that many people believe that they are always compliant while others are the “problem.”

Several additional themes were identified throughout the interviews. All interviewees noted that there was incentive for both the clinical staff and the patients to reduce the number of INR tests being ordered. For staff members, this lead to overall reductions in work load. For patients, less frequent blood draws were often seen as a reward for prior good INR control. Work load issues were particularly salient for the interviewees. They acknowledged that increased work load in the short term, such as having to spend extra time doing patient education or documentation to “enroll” a patient in the less frequent INR testing protocol, was inversely related to their likelihood to decrease INR testing frequency for patients with stable warfarin control. They acknowledged that this was somewhat counter-intuitive as less frequent INR testing decrease work load in the long term, but that short-term work load increases are a significant barrier. Finally, when asked about how anticoagulation clinic staff identify eligible patients for less frequent INR testing, staff members were able to identify their clinic’s process for determining eligibility. However, there was always heavy emphasis on the patients that were not eligible (e.g., patients on home INR testing or patients with ventricular assist devices). This focus on identifying patients not eligible, rather than eligible, may indicate some level of staff reluctance to reduce the INR testing frequency for patients even when patients would otherwise qualify based on stable warfarin control. Equally, it may reflect some challenges that providers have in identifying eligible patients at the point of care.

**Fig. 1:**
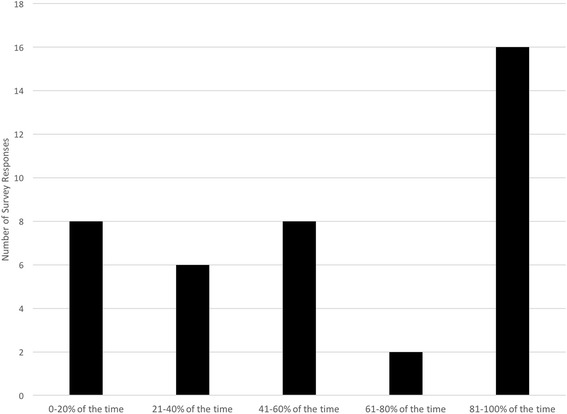
Self-reported Frequency of Extended INR Testing for Stable Warfarin Patients

A brief, 10-question survey was completed by 40 of the 62 invited nurse and pharmacist staff members (65% completion rate). Most respondents (*n* = 21; 54%) had more than 5 years of anticoagulation clinic experience. The majority of respondents (*n* = 32; 80%) were anticoagulation nurses while the remaining (*n* = 7; 18%) were pharmacists or did not report their clinical training (*n* = 1; 2%). Data on survey non-responders was not available. When asked to estimate a percentage of eligible patients who received a less frequent INR testing in the last 6 months, 16 (40%) selected 81–100% of the time while only 8 (20%) selected 0–20% of the time (Fig. 1). When asked to estimate how many of the prior 10 eligible patients received less frequent INR testing, the mean response was 7.8 (SD 2.4). Eighteen (45%) of respondents indicated that they always review a patient’s chart for less frequent INR testing eligibility while 16 (40%) reported doing this almost every time. A majority (*n* = 24; 60%) agreed that a flag in the electronic medical record would be very helpful in highlighting potentially eligible patients for less frequent INR testing. Twenty-four (60%) of respondents agreed that periodic, personalized feedback on INR testing frequency would be helpful. When asked about the number one barrier to routinely reducing INR testing frequency for eligible patients, survey responders frequently noted time constraints, need for additional or non-standardized documentation, staff forgetfulness, concerns for elderly patients, and patients requesting to stay with shorter intervals.

Based on audit data from the MAQI^2^ registry of patients on chronic warfarin therapy, less frequent INR testing was offered to 580 of 921 (63.0%) eligible MAQI^2^ patients over the first 6 months of 2016. The survey responders indicated that they offered less frequent INR testing to eligible patients 56% of the time (CI 46–66%).

## Discussion

This mixed-methods study of anticoagulation clinic nurses and pharmacists highlights important considerations for any new de-implementation policy or clinical practice at the provider level. With regards to reducing the frequency of INR testing for patients with stable warfarin control, this includes easy access to INR lab trends with flags for potentially eligible patients, minimizing extra work or patient education that occurs when selecting the next scheduled INR testing date, and providing individualized feedback on appropriate INR testing frequency. The survey responses enhance the credibility and of the interview findings.

One other health system has published their experience with a similar de-implementation effort to reduce frequent INR tests among patients with stable warfarin control [16, 17]. However, they did not report on how frequently their staff scheduled the next INR test more than the standard 4 weeks into the future for eligible patients or the barriers and facilitators to their de-implementation effort. Within the five centers participating in our current project, a significant amount of education was provided to the anticoagulation staff by their medical directors prior to and during the roll out of this new practice of less frequent INR testing for patients with stable warfarin control. Continuous monitoring, however, demonstrated that a reduction in INR testing was limited. Further, staff training efforts only had modest impact on overall de-implementation effort. The current study highlights important barriers beyond “staff training” that likely contribute to below goal utilization rates and success at de-implementation of current practice patterns.

By using the TICD determinants of implementation, we identified specific barriers in addition to “provider knowledge” that likely influence provider behavior [7]. In the case of reducing unnecessary INR laboratory tests for patients with stable warfarin treatment, these align with “feasibility and accessibility”, “compatibility and effort”, “knowledge about a provider’s own practice”, “self-monitoring or feedback”, and “information system barriers” from the TICD determinants. Comparing the self-reported survey data to the actual reduction in INR testing finds reasonable accuracy in the self-reported data. However, the semi-structured interviewees were surprised to hear the actual rate of less frequent INR testing among eligible patients, perhaps suggesting that these were a somewhat biased group of selected anticoagulation staff members. In contrast to prior studies suggesting clinician overestimation of guideline-adherence practice, our study suggests that these clinicians either accurately or slightly underestimate the frequency with which they reduced INR testing frequency [18]. Still, providing personalized and timely feedback is important to inform staff of their performance and opportunities for improved patient care. While none of these barriers are novel or new to implementation researchers, they may not be universally considered by anticoagulation clinic directors who desire to implement local policy or procedural change. This was certainly the case within the five participating centers for this study.

In response to these findings, we are developing tools within our electronic health records to automatically screen for and flag patients who are likely eligible for less frequent INR testing, based on 4 months of continuously in range INR values. We anticipate that this information system change will help address concerns about difficulty reviewing all prior INR values easily and any unintended forgetfulness that can occur when staff are busy with many patient labs to review and act upon. We are also developing systems to provide more personalized feedback at each clinical site to individual anticoagulation clinic staff about their recent patients who were eligible for less frequent INR testing, and whether or not it was offered during the encounter. We continue our anticoagulation staff education efforts, reviewing utilization and safety data from all MAQI^2^ sites to address any staff concerns. We believe this may be critical for the 20% of survey respondents who reported rarely attempting to reduce INR testing frequency (Fig. 1). Finally, we are working to remove barriers to reducing INR testing frequency, including reducing the amount of extra patient education or clinical documentation that is needed for an anticoagulation nurse or pharmacist to begin using this process for eligible patients. Instead, we are focusing on improving the patient education material to highlight that INR testing intervals may be extended beyond 4 weeks once patients have established a very stable treatment regimen.

Our study has several important strengths, including the use of a mixed-methods design which allows for deeper understanding of potential practice barriers and breadth of representation through survey work. However, important limitations must be considered when interpreting these results. First, we were unable to interview all anticoagulation clinic staff, and therefore the themes identified through the interviews may not represent the entire spectrum of experiences. However, with the eight interviews performed, we felt that thematic saturation was reached, and that these findings were largely supported in the broader anticoagulation staff survey. Second, while we could assess barriers across five diverse anticoagulation clinics, each of these clinics is located in southern Michigan and participates in an insurer-sponsored quality improvement collaborative. Additionally, we were only able to interview a single pharmacist, limiting comparisons between nurses and pharmacists working in anticoagulation clinics. Finally, no formal triangulation analysis was performed. These may limit generalizability to all anticoagulation clinics in the United States of America or beyond.

## Conclusions

In summary, using a mixed-methods approach we identified key barriers and facilitators to a de-implementation effort to reduce frequent INR laboratory tests among patients with stable warfarin control in five anticoagulation clinics and their staff. Beyond providing staff education, leveraging information system notifications, reducing additional work load burden for participating patients and anticoagulation clinic staff, and providing personalized feedback to anticoagulation clinic staff appear to be key strategies that may improve adoption and utilization of a policy that requires de-implementation of an existing practice. Evaluating the effect of these interventions and the relationship of these barriers to other anticoagulation clinic policy changes should be explored in future work.

## Additional file

Additional file 1: Semi-structured Interview Guide. (DOCX 20 kb)

## Abbreviations

INR: International Normalized Ratio of the prothrombin time
MAQI^2^: Michigan Anticoagulation Quality Improvement Initiative
TICD: Tailored Implementation for Chronic Disease
