# Supplementary material for: Barriers and facilitators to reducing frequent laboratory testing for patients who are stable on warfarin: a mixed methods study of de-implementation in five anticoagulation clinics
Source: Implement Sci. 2017 Jul 14;12:87. doi: 10.1186/s13012-017-0620-x (PMC5513354; doi:10.1186/s13012-017-0620-x)
Supplement: Additional file 1: — Semi-structured Interview Guide. (DOCX 20 kb) [file 13012_2017_620_MOESM1_ESM.docx]

**Additional file 1**

| **Question** | **Probes** | **TICD Domains and Determinants of Practice[1]** |
| --- | --- | --- |
| *Process Steps, Barriers and Facilitators* | | |
| How do you currently determine when a patient needs their next INR checked? | - Walk me through each step - How do you assess INR stability? - What changes for INR testing interval >4wks? | - Individual Health Professional Factors/Professional Behavior/Nature of Behavior |
| How easy is it for you to set the next INR testing interval beyond 4 weeks? | - What resources do you need to extent the INR testing interval for stable patients? - Are the necessary resources available? - What could be done to make it easier to set the next INR date beyond 4 weeks when appropriate? - How much extra effort is required for extended INR interval? - What contributes to the extra effort? - What could be done to reduce that extra effort? - Would reducing extra effort make you more likely to extend INR testing interval? | - Guideline Factors/Recommended Clinical Intervention/Accessibility of the Intervention - Incentives and Resources/Availability - Guideline Factors/Recommended Behavior/Effort |
| Are patients open to an extended INR testing interval? | - How does a patient’s willingness to extend their INR testing interval impact your ability to adhere to this policy? | - Patient Factors/Patient Needs - Patient Factors/Patient Preferences |
| When you extend a patients INR testing interval, do they usually comply? | - What reasons do they give for not complying? - How does this impact your willingness to extend a future patient’s INR testing interval? | - Patient Factors/Patient Behaviors |
|  | | |
| *Knowledge, Accessibility and Attitudes of the Policy* | | |
| When thinking about a patient’s eligibility for an extended INR testing interval, can you tell me what qualifies a patient as “stable”? | - Can you show me the policy? - Are there details of the policy that you’re not aware of? - Are there portions of the policy that are not clear or easy to understand and apply? - Are there details of the policy that you need more education about? | - Individual Health Professional Factors/Knowledge and Skills/Domain Knowledge - Guideline Factors/Recommendation/Clarity - Guideline Factors/Recommendation/Accessibility of Recommendation - Individual Health Professional Factors/Knowledge and Skills/Awareness and Familiarity with the Recommendation |
| How easy is it for you to identify stable warfarin patients who might be eligible for an extended INR testing interval? | - What resources do you need to identify these stable warfarin patients? - Are the resources available? - What could be done to make it easier to identify qualified patients? | - Guideline Factors/Recommended Clinical Intervention/Accessibility of the Intervention - Incentives and Resources/Availability of Necessary Resources |
|  | | |
| *Barriers and Facilitators (Part I)* | | |
| What would be an incentive for you or another ACS staff to identify a stable patient and extend their INR testing interval? | - Financial - Non-financial - Barriers | - Incentives and Resources/Availability of Necessary Resources - Incentives and Resources/Financial Incentives and Disincentives - Incentives and Resources/Nonfinancial Incentives and Disincentives |
| What incentives do the patients have to extend their INR testing interval? | - Financial - Non-financial - Barriers | - Incentives and Resources/Availability of Necessary Resources - Incentives and Resources/Financial Incentives and Disincentives - Incentives and Resources/Nonfinancial Incentives and Disincentives |
| How does the EHR or computer system help you find stable patients and extend their INR testing interval? | - Facilitators - Barriers | - Incentives and Resources/Information system |
| Do you have the necessary support to adhere to the extended INR testing interval policy? | Would these be helpful? Why or why not?   - Checklists - Patient information - Decision aids - Decision support tools - Additional clinical supervision - What additional skills do you need? | - Incentives and Resources/Assistance for Clinicians - Individual Health Professional Factors/Knowledge and Skills/Skills Needed to Adhere |
| Does continuing education impact your ability to extend the INR testing interval? | - How is continuing education best delivered for you? | - Incentives and Resources/Continuing Education System - Individual Health Professional Factors/Knowledge and Skills/Learning Style |
|  | | |
| *Outcomes and Follow Up* | | |
| How often do you think that you are extending the INR testing interval for eligible patients? | - More or less than colleagues? | - Individual Health Professional Factors/Knowledge and Skills/Knowledge about Own Practice |
| Do you think feedback on your success with extended INR testing would be beneficial? | - Clinic level feedback - Individual level feedback - Timeliness - What specific feedback would be most helpful? | - Individual Health Professional Factors/Professional Behavior/Self-Monitoring or Feedback |
|  | | |
| *Optional Additional Questions* | | |
| Can you tell me about the evidence supporting the use of an extended INR testing interval for stable warfarin patients? | - Do you believe the evidence applies to your patients? - How does your personal or clinic’s experience influence your confidence in the published data? | - Guideline Factors/Recommendation/Quality of Evidence Supporting the Recommendation |
| Who created the extended INR testing policy? | - In your eyes, do they have credibility? | - Guideline Factors/Recommendation/Source of the Recommendation |
| What do you expect to happen when you recommend an extended INR testing interval to a stable warfarin patient? | - Will the patient agree/disagree? - Will they comply? - Will the follow up INR be in-range or out-of-range? - Is this a safe practice? Are you concerned this puts them at risk of complications? | - Individual Health Professional Factors/Knowledge and Skills/Expected Outcome |
| Do you see any benefits when you extend the INR testing interval for stable patients? | - Benefits to the patient? - Benefits to yourself? - Benefits to the ACS as a whole? - Do you ever see harms? | - Individual Health Professional Factors/Recommended Behavior/Observability |

1. Flottorp SA, Oxman AD, Krause J, Musila NR, Wensing M, Godycki-Cwirko M, Baker R, Eccles MP: **A checklist for identifying determinants of practice: a systematic review and synthesis of frameworks and taxonomies of factors that prevent or enable improvements in healthcare professional practice.** *Implement Sci* 2013, **8:**35.
